# Literature mining for context-specific molecular relations using multimodal representations (COMMODAR)

**DOI:** 10.1186/s12859-020-3396-y

**Published:** 2020-10-26

**Authors:** Jaehyun Lee, Doheon Lee, Kwang Hyung Lee

**Affiliations:** 1 Department of Bio and Brain Engineering, Korea Advanced Institute of Science and Technology, Daejeon, South Korea; 2 Bio-Synergy Research Center, Daejeon, South Korea

**Keywords:** Biological context, Literature mining, Natural language processing, Representation learning

## Abstract

**Abstract:**

Biological contextual information helps understand various phenomena occurring in the biological systems consisting of complex molecular relations. The construction of context-specific relational resources vastly relies on laborious manual extraction from unstructured literature. In this paper, we propose COMMODAR, a machine learning-based literature mining framework for context-specific molecular relations using multimodal representations. The main idea of COMMODAR is the feature augmentation by the cooperation of multimodal representations for relation extraction. We leveraged biomedical domain knowledge as well as canonical linguistic information for more comprehensive representations of textual sources. The models based on multiple modalities outperformed those solely based on the linguistic modality. We applied COMMODAR to the 14 million PubMed abstracts and extracted 9214 context-specific molecular relations. All corpora, extracted data, evaluation results, and the implementation code are downloadable at https://github.com/jae-hyun-lee/commodar.

**Ccs concepts:**

• Computing methodologies~Information extraction • Computing methodologies~Neural networks • Applied computing~Biological networks.

## Background

Complex biological systems are known to comprise the coordination of molecular interactions and the relationship between molecules will consequently determine the behavior of the entire system. Molecular network models are often considered to be valuable for elucidating the organizing principles of biological systems and promoting public health. For example, biological networks are of pharmacological interest as an aid to the prediction of the side effects or multi-targeting drug efficacy.

In the pursuit to develop network models, biomedical researchers have increasingly depended on informatics resources which serve various patterns of molecular relations [1]. Yoon et al. had integrated pathway resources comprised of the relations between biological molecules and substantiated that information from various resources were sometimes contradictory [2]. For instance, one database supports that *a protein A INCREASES the activity of a protein B*, whereas another one supports that *the protein A DECREASES the activity of the protein B*. Yoon et al. partially attributed these discrepancies to the lack of the contextual information, which specified the biological circumstance of the molecular relations. As a solution, this study enhanced the resolution of context-free data and resolved the rate of the information conflict. That is to say, if *the protein A has a positive influence on the protein B in HEALTHY cases* while *negative in MELANOMA patients*, two augmented relations no longer are contradictory. The context types considered in this study include cell type, organ, disease, and drug.

dSysMap [3] and PinSnps [4] are examples of repositories of the protein interactions functionally perturbed by pathological mutations. These resources ensure a higher resolution of molecular interaction data mathematically structured from other public data resources by specifying genetic conditions. TIMBAL [5], 2P2Idb [6], and iPPI-DB [7] house not only protein-protein interactions (PPI) but also small molecules which are putatively druggable and have been proven to modulate associated protein interactant pairs. This context-specific information has been collected from the public databases (TIMBAL, 2P2Idb) or hand-curated from the biomedical literature (iPPI-DB). In other words, the aforementioned resources rely on laborious manual curation or other structured resources which have been manually prepared.

An enormous wealth of biomedical information resides in unstructured written languages such as journal articles, which has been unprecedentedly growing. To be more specific, nearly 30 million references are available in PubMed and has annually published more than one million papers. As the number of biomedical publications continues to grow, such an exponentially growing volume of literature has become infeasible to be structured. Thereby, the gap between published knowledge and well-tailored information in databases has been widening.

## Previous work

To assist this situation, several text mining efforts [2, 8–10] have been attempted. These approaches identified the contextual information corresponding to molecular pairs of interaction from the specific publications reporting the given molecular interactions. Poon et al. and Yu et al. used the specific types of the medical subject headings (MeSH) [11] terms annotated on each PubMed abstract provided by MEDLINE whereas Lee et al. and Yoon et al. annotated every context mention recognized in the abstract text. While these efforts are capable to efficiently augment the given information, the co-occurrence-based extraction generally suffers from low precision due to its greedy behavior. Machine learning offers a much more attractive alternative by effectively automating the elaborate pattern engineering. Furthermore, natural language processing (NLP) based on the machine learning approach automatically enables to analyze textual sources and streamlines the extraction of facts and knowledge to support biomedical database curation.

Conventional machine learning frameworks for biomedical relation extraction often rely on linguistic information, such as word n-gram or syntactic dependency, directly derived from literature text. Quan et al. [12] employed word embedding models based on word n-gram proximity, also known as distributed representations to capture linguistic patterns from sentences and predict drug-drug interactions (DDI). PPI. Zhao et al. [13] refined the n-gram model which numerically represents words by syntactic dependency information achieved by deep parsing and predicted DDI. These endeavors focused on linguistic information to analyze sentences and extract the planar information of interactions between two entities. On the contrary, the information of interest in the present paper, i.e., context-specific molecular relation, includes the contextual modulator of two molecular entities as well as the relation between two molecules. Therefore, the extraction task for such a meta-relation may be accelerated by extra features in addition to canonical linguistic information based on word n-gram and syntactic dependency. In the biomedical domain, well-structured and comprehensive knowledge graphs such as MeSH or unified medical language system (UMLS) [14] contain relational knowledge in forms of the knowledge triplet represented as *<subject, predicate, object>*. The biomedical domain information from these knowledge resources can play a complementary role in the holistic representation of the unstructured text.

**Fig. 1:**
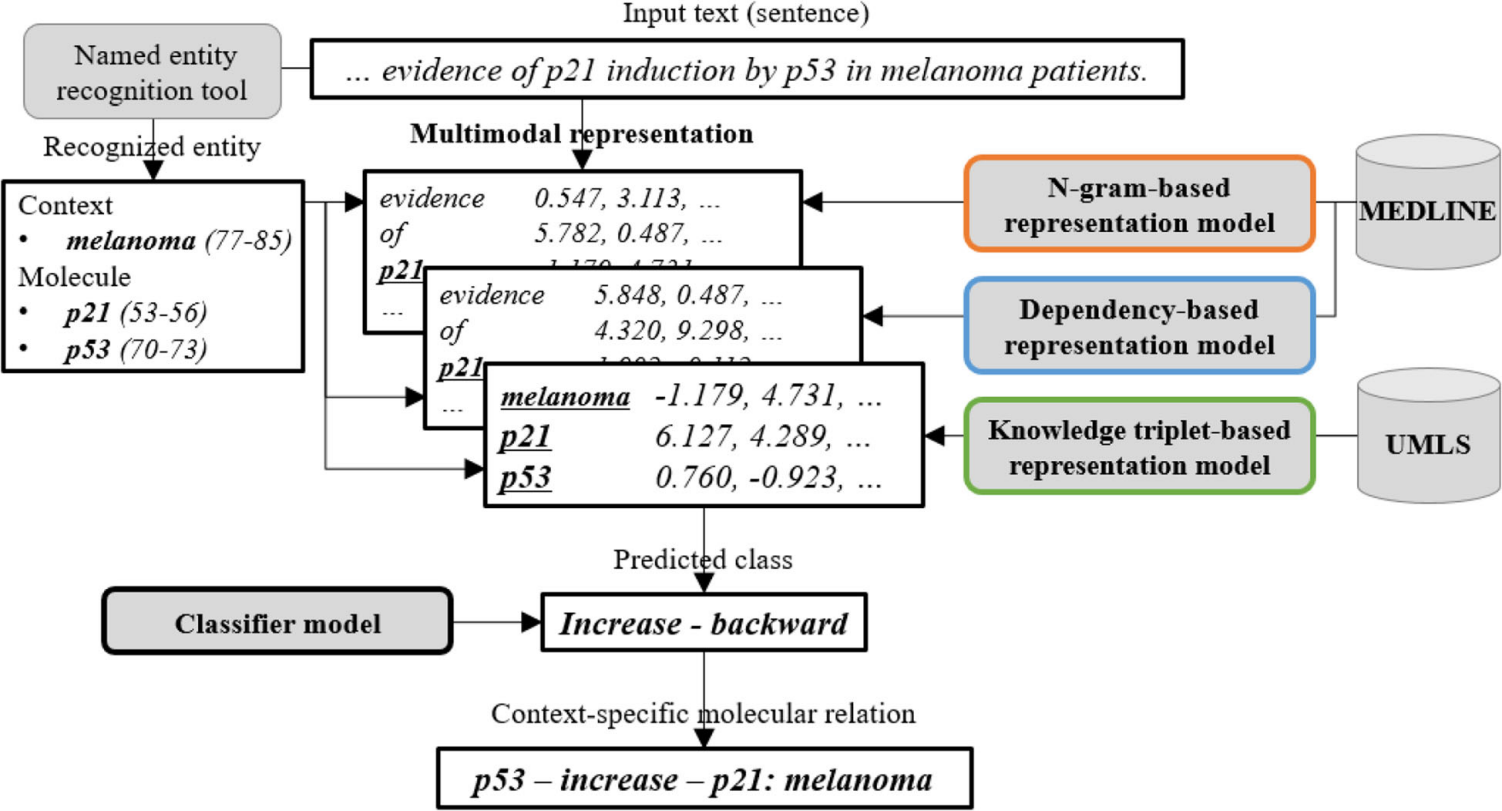
The overall process of COMMODAR

In the present paper, we propose a machine learning approach COMMODAR, which extracts context-specific molecular relations from biomedical literature. COMMODAR cooperatively uses three discriminative features from multiple modalities, which includes linguistics (word n-gram and syntactic dependency) and biomedical knowledge (knowledge triplet). The overall process of COMMODAR is illustrated in Fig. 1.

## Method

The specification of the information to be extracted from the literature is summarized below.

(i) Molecular type: Gene, Gene product
(ii) Context type: Disease
(iii) Relation type: Increase, Decrease, Regulate (sign unknown), Binding (sign and direction unknown)

We considered the extraction of the context-specific molecular relations as a sentence classification problem. To solve the classification problem, we used a supervised-learning paradigm based on the ground-truth data.

### Preprocessing

#### Abstract collection and sentence preparation

We downloaded PubMed raw files in an XML format and retrieved abstract texts published until 2016 resulting in 14,891,354 abstracts. Collected abstracts were segmented sentence-by-sentence by GENIA Sentence Splitter [15]. All characters were lowercased and a set of punctuation marks (parentheses, comma, slash, hyphen, exclamation mark, question mark, and quotation mark) was spared instead of being eliminated due to following syntactic parsing. The spared punctuation marks and general words were separated by white space, e.g., *(general words*). Special characters other than the aforementioned punctuation marks were eliminated.

#### Name entity recognition (NER) and normalization

To recognize molecular entities in the sentence, BANNER [16], the most widely used NER tool for gene, gene product, and disease entities, was exploited. Afterward, UMLS terminology browser API assigned UMLS concept unique id (CUI) in the version of 2018AB with the ‘Exact Match’ search type to the recognized entities for term normalization. The CUI was specified by UMLS semantic types as listed in the Table A1 (See Additional file 1) according to UMLS semantic group: Genes & Molecular Sequences and Chemicals & Drugs for molecular entities whereas Disorder for context entities [17]. On one hand, the semantic type Cell Component (celc) in the semantic group Anatomy was included to cover protein complex entities. On the other hand, the semantic type Finding (fndg) in the semantic group Disorder was excluded because Finding indicates not the disease concept but the discovery or diagnosis of the disease. This term normalization process for the recognized entities establishes the bridge of multimodality so that the given textual source can be represented by both linguistic and biomedical knowledge.

### Representation model

#### N-gram-based word embedding

N-gram-based word embedding is the distributed representation of word to capture the semantic information in the unsupervised manner. After sentence segmentation and tokenization of collected abstracts described in the previous section, the skip-gram model [18] was employed to train the word representation. The n-gram-based word embedding model was implemented with the open-source Python library GenSim [19]. The embedding dimension size, window size, iteration, learning rate, subsampling, and negative sampling size were 200, 16, 50, 0.05, 1e-5, and 10, respectively. These hyper-parameters were determined according to Chiu et al.’s work especially for biomedical NLP and the rest remained the default [20]. As a result, 1,960,501 of the vocabulary was totally yielded after the training procedure.

#### Dependency-based word embedding

In contrast to n-gram-based word embedding defining a target word by the neighbor words of the target word in sentences, dependency-based word embedding defines a word by its syntactic dependency [21]. Syntactic dependency refers to the syntactic relations between words in the sentence. For example, a verb *saw* has a dependency on its direct objective *cat* in a sentence *I saw a cat* and it can be represented as *saw-cat/dobj*. Dependency-based word embedding has been known to have different properties from n-gram-based word embedding and to capture the functional properties of words [22]. Intuitively speaking, n-gram-based word embedding considers the words co-occurring in the window of a pre-defined size with the target words in sentences, while dependency-based word embedding considers the words functionally related to the target words regardless of the distance. Syntactically parsed results distributed by Hakala et al. consist of 8,934,832 abstracts, 55,092,436 sentences, and 55,416,433 words [23]. The embedding dimension was 200 and the rest of hyper-parameters was stuck with the default.

#### Knowledge triplet-based concept embedding

The UMLS Semantic Network stratifies biomedical concepts in the UMLS Metathesaurus and presents useful relations between the sets of these concepts. The nodes and edges in the network are the semantic types and the relations between semantic types, respectively and 2018AB version offers 133 semantic types, 54 relation types, and totally 6105 relations. To capture global relations and transitional characteristics between semantic types, we employed ConvKB, a knowledge graph embedding model based on convolutional neural networks (CNN) [24]. ConvKB concatenates subject, predicate, and object vectors into an *m* × 3 matrix, then feeds it into a CNN with the 1 × 3 filters, where *m* is the dimension of the embedding vector. Thus, ConvKB is a generalized version of the transitional knowledge graph embedding framework TransE [25]. The dimension of the embedding vector and the learning rate were set as 10 and 0.001, respectively, while the rest of the hyper-parameters as the default.

### Classification model

#### Corpus

Unfortunately, to our knowledge, any labeled sentence set of context-specific molecular relations has never been developed for the public purpose. Therefore, we considered a transfer learning scheme to manually generate a small volume of completely labeled sentences and additionally leverage the relatively sizable corpora for related tasks [26]. Partially matched ground-truth data, i.e., the annotation of context-free molecular relations, includes Genia Task (GE11, GE13) and Pathway Curation (PC11, PC13) distributed by the BioNLP Shared Task (BioNLP-ST) workshop [27, 28]. The BioNLP-ST workshop is a decade-long series of community-wide efforts toward structural literature mining in the biomedical domain. Besides those manually annotated corpora, EVEX also incorporates context-free relational information computationally extracted by a machine learning framework, TEES [29]. The detailed preprocessing procedure to prepare context-free corpora is illustrated in the Appendix A.1 (See Additional file 1). The number of class labels is totally eight for both context-free and context-specific corpora: Increase-forward, Increase-backward, Decrease-forward, Decrease-backward, Regulate-forward, Regulate-backward, Binding, and False. The class labels are identical but there are differences in the implication of the labels and the sentences involved in each label between context-free and context-specific corpora. Sentences in context-free corpora describe the generic relations between molecules without any contextual specification, i.e., disease condition, while sentences in context-specific corpora describe the context-specific relations. Thus, class labels of context-free corpora refer to generic relations whereas those of context-specific corpora to context-specific relations. The final volume of the corpora is enumerated in the Table A4 (See Additional file 1).

#### Model architecture

**Fig. 2:**
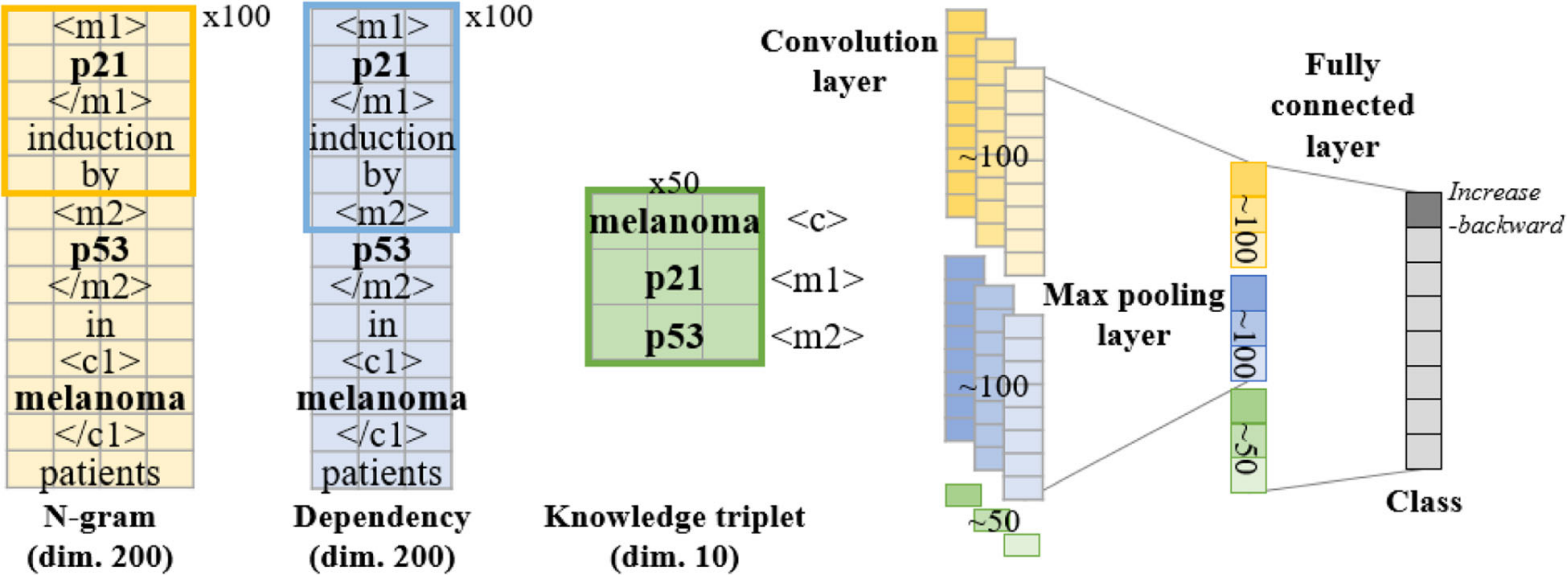
The architecture of MGNC-CNN

To fully capture the relational information implicitly expressed in a sentence, we conducted automated feature learning with CNN obviating manual feature engineering. The convolutional layers and the pooling layers in CNN extract local features in a sentence and merge local feature patterns, respectively. In addition, the multi-group norm constraint CNN (MGNC-CNN) architecture independently extracts features from the multiple embedding sets and generates a final feature vector by concatenating the extracted high-level features at the penultimate layer [30]. MGNC-CNN shows a higher degree of freedom than other CNN architectures accommodating multiple embeddings such as multi-channel CNN [12] because MGNC-CNN can manipulate embeddings with various vector sizes and regularization strategies. An architecture of MGNC-CNN we equipped for the relation classification is represented in Fig. 2. For relation classification, relation entities such as molecule and context keywords were marked along with entity tagging features and the detailed description was illustrated in the Appendix A.2 (See Additional file 1) [31].

According to the transfer learning scheme, the parameters in MGNC-CNN were initialized by pre-training with context-free source corpora established by both the manual review and computational inference and then, fine-tuned by the context-specific target corpus which had been manually generated for the present study. The filters with the length of 21, 22, and 23 were applied for the linguistic representation (both n-gram-based and dependency-based embeddings) and the width was dependent on the embedding vector size, therefore, 200. Because the dimension of the filter for the biomedical knowledge representation (knowledge triplet-based embedding) was 3 × 10 which was congruent with the knowledge matrix, the convolution operation over the knowledge matrix was applied once without any stride. The numbers of filters were 100, 100, and 100 for the linguistic representation and 50 for the biomedical knowledge representation, respectively. We set the learning rate of 0.001, the drop-out probability of 0.5, ADAM optimizer, and early stopping (less than 10 epochs for all cases) for both pre-training and fine-tuning. The size of the mini-batches was set by 50 and 5 for pre-training and fine-tuning, respectively.

#### Post-processing

After predicting class labels of putative context-specific relations described in the sentences, we analyzed the frequency of extracted relations to estimate the significance. Based on the assumption that more frequently reported the relation is at the research articles, the more significant the relation can be considered, the rare relations reported less than the empirical threshold were discarded. The empirical threshold was determined as six times, the frequency of the relation which was the top 1% (0.01) in the frequency distribution of the entire extracted relations. The relations reported more than 14 times were grouped by the sequence of three CUIs of one contextual and two molecular entities. The conflict of relation classes in a group was resolved by voting, in other words, the majority of relation classes. In case of the tie, the relation classes were abstracted, for example, Increase and Decrease with the same direction result in Regulation whereas relation classes with contradictory directions result in Binding.

## Result

### Performance of the classification model

**Fig. 3:**
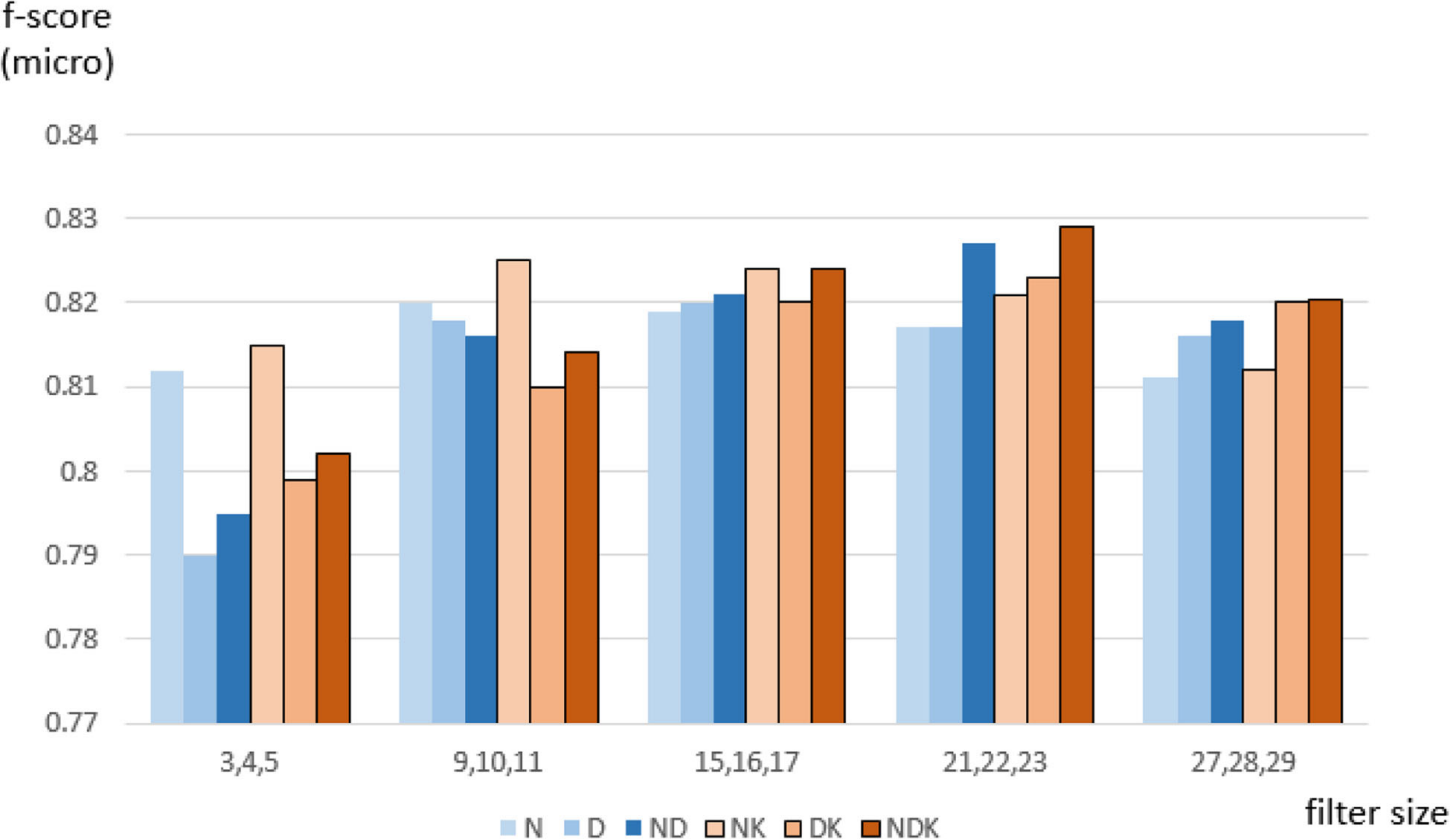
Performance comparison across various embedding combinations and filter sizes

The performance of MGNC-CNN model was evaluated in the micro-average f-score because the class distribution in the context-specific corpus was highly skewed. The context-free and context-specific corpora were respectively separated into training and test datasets (2:1) for 3-fold cross-validation. Figure 3 shows the result of the ablation analysis across various representation combinations and filter sizes. N, D, and K stand for n-gram-based, dependency-based, and knowledge triplet-based representations, respectively. The models using the knowledge triplet-based representation illustrated in brown colors mainly boosted the performance by adding biological background knowledge about relational entities. Finally, the model using three different representations with filter size 21, 22, and 23 outperformed other representation combinations and filter size ranges. The statistical analysis about the putative optimal filter size is illustrated in the Appendix A.3 (See Additional file 1). The model solely based on the knowledge triplet representation was excluded for the lack of the linguistic information spread across the sentence.

### End-to-end inspection

We applied the context-specific model to the preprocessed unlabeled sentences and inspected 177 randomly sampled sentences with predicted labels to estimate the end-to-end reliability of the proposed framework. 73 sentences were correctly predicted, while 104 sentences were assigned wrong labels with various circumstances. 67 out of 73 correctly predicted sentences were False sentences and the other 6 implied the specific relations. 30 out of 67 true negative sentences were describing valid molecular relations while mentioned context words were invalid. The model based on the co-occurrence assumption is likely to classify these False sentences as valid while COMMODAR was enabled to exclude. Furthermore, these correctly classified False sentence support that COMMODAR is not fully biased on the pre-trained model which extracts context-free molecular relations and is expected to classify these False sentences as valid. 80 False sentences out of 104 failures incorrectly labeled as specific relations, the 7 relational sentences were predicted to the wrong relation types or the opposite directions, and the rest 17 sentences were false negative. 80 False sentences include 13 with NER errors, 48 without any relation between molecular entities, 19 describing experimental designs or objectives rather than unraveled facts or results. Because of the false discovering behavior of the classification model extracting relations from False sentences, the conservative threshold, 0.01 was applied in the post-processing step.

### Repository of context-specific molecular relations

After large-scale extraction and post-processing, we yielded 9214 context-specific molecular relations (Binding 4864, Regulation 1475, Increase 1448, and Decrease 1427) previously reported in the literature. The most frequent contextual concept was Neoplasm (C0027651) and the 2030 relations were extracted to be specific to Neoplasm. The example sentences for the end-to-end inspection and all the extracted relations are downloadable in https://github.com/jae-hyun-lee/commodar.

## Discussion

COMMODAR focuses on the context type of the disease in the proposed research. However, it can be technically extended to deal with alternative context types such as medication or anatomy if only relevant NER tools and a small volume of the completely labeled sentence set are available. Thus, it can be utilized to build and maintain databases containing various context types for molecular relations. Moreover, molecular relations extracted by COMMODAR can resolve the protein in the isoform level thanks to the entity normalization according to UMLS which distinguishes protein isoforms. This fine resolution provides the extracted context-specific molecular relations with versatility.

COMMODAR, however, has some limitations to be noted. Firstly, it requires the entity normalization step which is mostly unnecessary for relation extraction. Two molecule entities and one context entity should be normalized with UMLS CUI to utilize background knowledge from the knowledge graph, i.e. UMLS as well as linguistic information. Secondly, COMMODAR showed the false discovering behavior by producing 80 false positive out of 177 sentences and we proposed the frequency-based post-processing procedure. Nevertheless, it is partially attributed to the nature of the given task, i.e. highly skewed true/false balance and the conventional co-occurrence-based models may well produce a larger number of false positives.

## Conclusion

We have proposed COMMODAR, a literature mining framework for context-specific molecular relations using multimodal representations. COMMODAR can utilize multiple representations from multiple modalities so that comprehensive information from various resources can cooperate to analyze the unstructured text. The superiority of multimodal information in the relation extraction task was substantiated by the outperformance of the MGNC-CNN model using both linguistic and biomedical knowledge representations. The expansibility of COMMODAR enables the biomedical database curators to adopt various state-of-the-art and off-the-shelf embedding models appropriate to the characteristics of the data resources.

## Supplementary information

**Additional file 1.** Table A1 Semantic types for molecular and context entities. Table A2 Rule-based decomposition of nested events. Table A3 Rule-based conversion from event trigger types to relation classes. Table A4 The statistics of corpora. A.1 Conversion from events to relations. A.2 Entity tagging feature (ETF). A.3 The distribution of the token distance between entities in the context-specific corpus. Figure A1 The distribution of the token distance between entities.

## Data Availability

The datasets generated and analyzed during the current study are available in the GitHub repository, https://github.com/jae-hyun-lee/commodar.

## Abbreviations

BioNLP-ST: BioNLP Shared Task
celc: Cell Component
CNN: Convolutional neural networks
CUI: Concept unique id
DDI: Drug-drug interactions
fndg: Finding
MeSH: Medical subject headings
MGNC-CNN: multi-group norm constraint convolutional neural networks
NER: Name entity recognition
NLP: Natural language processing
PPI: Protein-protein interactions
UMLS: Unified medical language system
