# Supplementary material for: Literature mining for context-specific molecular relations using multimodal representations (COMMODAR)
Source: BMC Bioinformatics. 2020 Oct 26;21(Suppl 5):250. doi: 10.1186/s12859-020-3396-y (PMC7586695; doi:10.1186/s12859-020-3396-y)
Supplement: Supplementary file 1 — Additional file 1. Table A1 Semantic types for molecular and context entities. Table A2 Rule-based decomposition of nested events. Table A3 Rule-based conversion from event trigger types to relation classes. Table A4 The statistics of corpora. A.1 Conversion from events to relations. A.2 Entity tagging feature (ETF). A.3 The distribution of the token distance between entities in the context-specific corpus. Figure A1 The distribution of the token distance between entities. [file 12859_2020_3396_MOESM1_ESM.pdf]

A APPENDICES

Table A1 Semantic types for molecular and context entities

Entity type	Semantic group	Semantic Type	ID
Molecule	Genes & Molecular Sequences	Amino Acid Sequence	T087
		Carbohydrate Sequence	T088
		Gene or Genome	T028
		Molecular Sequence	T085
		Nucleotide Sequence	T086
	Chemicals & Drugs	Amino Acid, Peptide, or Protein	T116
		Antibiotic	T195
		Biologically Active Substance	T123
		Biomedical or Dental Material	T122
		Chemical	T103
		Chemical Viewed Functionally	T120
		Chemical Viewed Structurally	T104
		Clinical Drug	T200
		Element, Ion, or Isotope	T196
		Enzyme	T126
		Hazardous or Poisonous Substance	T131
		Hormone	T125
		Immunologic Factor	T129
		Indicator, Reagent, or Diagnostic Aid	T130
		Inorganic Chemical	T197
		Nucleic Acid, Nucleoside, or Nucleotide	T114
		Organic Chemical	T109
		Pharmacologic Substance	T121
		Receptor	T192

Entity type	Semantic group	Semantic Type	ID
		Vitamin	T127
	Anatomy	Cell Component	T026
Context	Disorders	Acquired Abnormality	T020
		Anatomical Abnormality	T190
		Cell or Molecular Dysfunction	T049
		Congenital Abnormality	T019
		Disease or Syndrome	T047
		Experimental Model of Disease	T050
		Injury or Poisoning	T037
		Mental or Behavioral Dysfunction	T048
		Neoplastic Process	T191
		Pathologic Function	T046
		Sign or Symptom	T184

Table A2 Rule-based decomposition of nested events

Input	Output	Example
Pos(Cause, Theme: Pos)	Pos(Cause, Theme)	BRs induce accumulation of BZR1 protein
Neg(Cause, Theme: Neg)		E2 prevented downregulation of p21
Pos(Cause, Theme: Reg)	Reg(Cause, Theme)	PKS5 mediates PM H +- ATPase regulation
Reg(Cause, Theme: Pos)		CaM regulates activation of HSFs
Reg(Cause, Theme: Reg)		PDK1 is involved in the regulation of S6K
Neg(Cause, Theme: Reg)		GW5074 prevents this effect on ENT1 mRNA
Reg(Cause, Theme: Neg)		The effect of hCG in downregulating ER beta
Pos(Cause, Theme: Neg)	Neg(Cause, Theme)	DtRE is required for repression of CAB2
Neg(Cause, Theme: Pos)		BIN2 negatively regulates BZR1 accumulation

Table A3 Rule-based conversion from event trigger types to relation classes

Trigger type	Class
Activation, Gene_expression, Positive_regulation, Transcription, Translation	Increase
Degradation, Inactivation, Negative_regulation	Decrease
Regulation	Regulation
Binding	Binding

Table A4 The statistics of corpora

	Context-free		Context-specific
	By human curators	By TEES (Björne 2014)	
False	13,650	-	686
Increase	1,485 (1,036 / 449)	12,930 (9,008 / 3,922)	126 (89 / 37)
Decrease	613 (435 / 178)	3,155 (2,662 / 493)	82 (44 / 38)
Regulation	1,640 (1,124 / 516)	5,090 (3,517 / 1,573)	109 (79 / 30)
Binding	1,220	119,753	81
Total	18,608	140,928	1,084

(forward / backward)

A.1 Conversion from events to relations

Genia Task, Pathway Curation corpora, and EVEX database deliver the annotation of the molecular events, which consist of molecules and their event trigger words. For example, in the case of the phrase positive regulation of Rad53, positive regulation is a trigger and Rad53 is a molecular theme of the trigger—Pos(Theme: Rad53). In another case of positive regulation by E3B1, positive regulation is a trigger and E3B1 is a molecular cause of the trigger—Pos(Cause: E3B1). The composition of these event units can represent a molecular relation, for example, positive regulation of Rad53 by E3B1 represents that E3B1 is a molecular modulator of Rad53 in a positive way—Pos(Cause: E3B1, Theme: Rad53) (Van Landeghem, Hakala et al. 2012). By this rule-based procedure, we collected plenty of labeled sentences delivering context-free molecular relations. Moreover, the decomposition rules for nested events, e.g., Pos(Theme:Pos(Theme:Rad53)) and the conversion rules from event triggers in original corpora to relation classes in the present task are elucidated in the Table A2 and Table A3 according to Van Landeghem et al. and Yoon et al., respectively. Events with unlisted trigger types were not considered and predicted events with ‘very high confidence’ and ‘high confidence’ were collected from EVEX.

A.2 Entity tagging feature (ETF)

The following phrase is an example of ETF: *provide the first solid evidence that <m1>p21</m1> induction by <m2>p53</m2> during a <c> DNA damage </c> - induced* (PMID: 15735718). As shown above, six

ETFs, *i.e.*, <m1>, </m1>, <m2>, </m2>, <c>, and </c>, specify where the entities of interest start and end by being regarded as six additional tokens in the given sentence. The embedding vectors of six ETFs were randomly initialized and had the identical embedding dimension as other words. Additionally, every sentence should be zero-padded in the front and the rear sides as long as the length of the longest sentence in the corpus since CNN take the vectors of the uniform length. Therefore, the sentence s was converted to the word sequence of the size $l_{max} + 6$, where l_{max} denotes the length of the longest sentence in the corpus. The vector representation of a sentence (or a word sequence) S is formulated as follows:

$$S = [x_1, x_2, \dots, x_n]$$

where, n is the length of the sequence including six ETFs, *i.e.*, $l_{max} + 6$ and $x_n \in \mathbb{R}^{d_e}$.

A.3 The distribution of the token distance between entities in the context-specific corpus

'm1_m2', 'm1_c', and 'm2_c' in Figure A1 stand for the token distance in a sentence between two molecular entities, the first molecular and context entities, the second molecular and context entities, respectively, while 'max' is the maximum among aforementioned three distances. Five dark boxes highlight five filter size ranges used in the experiment. Figure A1 shows that the filter size 21, 22, and 23 covers the three entities in most of the sentences in the distribution of the token distance between entities. The performance attenuation over 27, 28, and 29 may be attributed to the growing number of parameters overwhelming the limited information of the context-specific corpus.

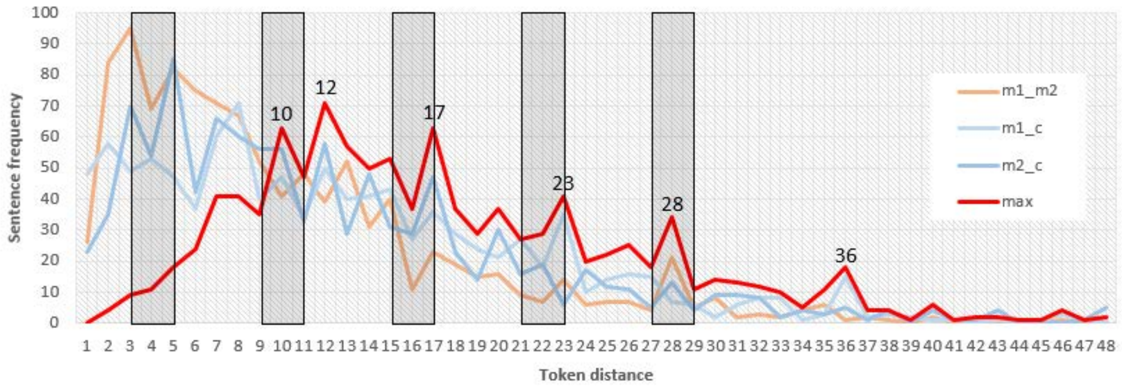

Figure A1 The distribution of the token distance between entities.
